# Perceptions and beliefs of community gatekeepers about genomic risk information in African cleft research

**DOI:** 10.1186/s12889-024-17987-z

**Published:** 2024-02-17

**Authors:** Abimbola M. Oladayo, Oluwakemi Odukoya, Veronica Sule, Ikenna Molobe, Tamara Busch, Babatunde Akodu, Wasiu L. Adeyemo, Lord J. J. Gowans, Mekonen Eshete, Azeez Alade, Waheed Awotoye, Adebowale A. Adeyemo, Peter A. Mossey, Anya E. R. Prince, Jeffrey C. Murray, Azeez Butali

**Affiliations:** 1 Department of Oral Pathology, Radiology and Medicine, College of Dentistry, University of Iowa, Iowa City, IA USA; 2 Iowa Institute of Oral Health Research, University of Iowa, Iowa City, IA USA; 3 Department of Community Health and Primary Care, College of Medicine, University of Lagos, Lagos, Nigeria; 4 Department of Operative Dentistry, College of Dentistry, University of Iowa, Iowa City, IA USA; 5 Department of Oral and Maxillofacial Surgery, University of Lagos, Lagos, Nigeria; 6 Komfo Anokye Teaching Hospital and Kwame Nkrumah University of Science and Technology, Kumasi, Ghana; 7 School of Medicine, Department of Surgery, Addis Ababa University, Addis Ababa, Ethiopia; 8 National Human Genomic Research Institute, Bethesda, MD USA; 9 Department of Orthodontics, University of Dundee, Dundee, UK; 10 College of Law, University of Iowa, Iowa City, USA; 11 Department of Pediatrics, University of Iowa, Iowa City, IA USA

**Keywords:** Community, Ethical issues, Return of results, Orofacial clefts, Africa, Health equity, Genomics research, Gatekeepers

## Abstract

**Background:**

A fundamental ethical issue in African genomics research is how socio-cultural factors impact perspectives, acceptance, and utility of genomic information, especially in stigmatizing conditions like orofacial clefts (OFCs). Previous research has shown that gatekeepers (e.g., religious, political, family or community leaders) wield considerable influence on the decision-making capabilities of their members, including health issues. Thus, their perspectives can inform the design of engagement strategies and increase exposure to the benefits of genomics testing/research. This is especially important for Africans underrepresented in genomic research. Our study aims to investigate the perspectives of gatekeepers concerning genomic risk information (GRI) in the presence of OFCs in a sub-Saharan African cohort.

**Methods:**

Twenty-five focus group discussions (FGDs) consisting of 214 gatekeepers (religious, community, ethnic leaders, and traditional birth attendants) in Lagos, Nigeria, explored the opinions of participants on genomic risk information (GRI), OFC experience, and the possibility of involvement in collaborative decision-making in Lagos, Nigeria. Transcripts generated from audio recordings were coded and analyzed in NVivo using thematic analysis.

**Results:**

Three main themes—knowledge, beliefs, and willingness to act—emerged from exploring the perspective of gatekeepers about GRI in this group. We observed mixed opinions regarding the acceptance of GRI. Many participants believed their role is to guide and support members when they receive results; this is based on the level of trust their members have in them. However, participants felt they would need to be trained by medical experts to do this. Also, religious and cultural beliefs were crucial to determining participants’ understanding of OFCs and the acceptance and utilization of GRI.

**Conclusions:**

Incorporating cultural sensitivity into public engagement could help develop appropriate strategies to manage conflicting ideologies surrounding genomic information in African communities. This will allow for more widespread access to the advances in genomics research in underrepresented populations. We also recommend a synergistic relationship between community health specialists/scientists, and community leaders, including spiritual providers to better understand and utilize GRI.

**Supplementary Information:**

The online version contains supplementary material available at 10.1186/s12889-024-17987-z.

## Background

To identify future research priorities and opportunities in human genomics, especially as they apply to human health and disease, the National Human Genome Research Institute (NHGRI) released a strategic vision document in 2020 [1]. Some of the foci highlighted in this document are achieving global diversity in genomics research by promoting the inclusion of diverse and underrepresented populations in large-scale genomic studies, improving access to genomics in healthcare, and maximizing the utility of genomics for all [1]. Also important is the discourse aimed at building, sustaining and improving a robust foundation for genomics, including improving genomic literacy across all sectors of society and empowering individuals to make well-informed decisions about genomic data [1].

In the context of African genomics research, realizing the benefits of genomic advances remains a work in progress. This goal is further complicated by the limited healthcare resources and a weaker genetics infrastructure [2, 3]. Low levels of genomic literacy have been reported among providers, patients, biomedical researchers and the public [4–7]. These barriers limit the incorporation of genomics into practice and patient’s understanding of the testing outcomes and decision-making. This culminates in reduced access to genomic medicine services, further exacerbating disparities in populations underrepresented in genomic research. Hence, there is a critical need to invest in efforts to prioritize these issues in African populations [8].

The quest to produce study findings better translated and applicable to all has led scientific entities and organizations to increase collaborations with community stakeholders. The hope is that community stakeholders can build on their relationships and motivate proposed participants. These influential stakeholders act as gatekeepers and are essential in gaining access to socially excluded populations in research [9]. However, these collaborations could pose some ethical challenges if these stakeholders are unfamiliar with the research goals and ethical challenges [10]. Several studies have reported the participation of gatekeepers—community, local, religious, informal, trade and traditional leaders, traditional birth attendants and healers—in successful health promotion programs such as reproductive health, polio and COVID-19 vaccination uptake, among others across the globe [11–15].

Regarding ethical issues associated with the delivery of genetic services in resource-limited settings, Zhong et al. in their systematic review, reported several ethical, social and cultural issues affecting genetic testing and counselling in Low and Middle-Income Countries (LMICs). These challenges include the difficulty of accessing genetic services, the social stigma associated with genetic conditions, the impact of cultural beliefs and practices on the uptake of information and understanding of genetic conditions, the need for support due to the psychosocial implications of genetic services, and the role of religion in accepting and utilizing genetic services [16]. This buttressed findings from other studies highlighting the need to comprehend genomic health information in culturally and linguistically diverse communities [4, 17–20]. This is in addition to the need for ethical and thoughtful methods in applying genomic services in LMICs [16, 21, 22]. In the absence of formal channels to obtain genomic health information, informal educational strategies may be required to enhance the genomic literacy of these heterogeneous groups, which points to the need for innovative approaches that are shared, assessed, and improved over time.

Gatekeepers are highly influential members of African communities, making them well-positioned to contribute towards a better understanding of genomic information [23]. However, this would require an in-depth knowledge of the socio-cultural dynamics influencing the perceptions of risk in these settings to prevent the perpetuation of biases in passing information across to community members [13, 24]. Community engagement (CE) provides an avenue to discuss the community’s needs while establishing a relationship between the researchers, communities and the research institutions and has been described as an essential component of conducting ethical biomedical research in ethnically diverse groups [25, 26]. As such, researchers have been advised to engage the participants and host communities before, during and post project completion to ensure that local perceptions are taken into consideration at every point in developing programs and interventions [25, 27, 28]. This was evident in the (CICP) model—Community Approach, Intermediate phase, Collaboration and Post‐research Cordiality of CE in genomic research proposed by Ogunrin et al. who proposed a stepwise approach to establishing ongoing collaboration with participants from minority communities[29].

Despite the breadth of work that has been conducted to address these ethico-legal issues, none of the existing research in limited resource settings, including sub-Saharan Africa, has focused on the experiences of community gatekeepers on genomic risk information (GRI), particularly in the context of orofacial clefts (OFCs). Orofacial clefts are common congenital anomalies with a varying incidence [30–33], however prevalence rates of 0.4–0.5 per 1000 births [34, 35] have been reported in Nigeria. Genetics play a significant role in the development of OFCs, nonetheless, environmental factors such as maternal nutrition, certain medications, and lifestyle choices have been implicated [36]. The experience of OFCs is such accompanied by a combination of physical, financial, mental and psychological burden and associated stigmatization [34], which poses unique challenges to individuals affected with cleft abnormalities and their families. Like, other LMICs, OFC management in Nigeria is also compounded by limited access to healthcare, inadequate prenatal care, and a limited awareness of the etiology of clefts and cleft care services [37]. As such, when gatekeepers can understand the nature and purpose of research and testing, they can help address misinformation around the risks and benefits of participation. They can also help address practices perpetuating stigma and discrimination related to research/ testing outcomes in the community [25].

Our study was designed to explore the opinions of community gatekeepers by conducting FGDs with ethnic, religious, community and traditional leaders. Questions were formulated based on previous literature on the subject, and we sought to fill the gap in knowledge about the perceptions of gatekeepers in a sub-Saharan African population concerning GRI in the presence of OFCs and their opinions and perceived role in weighing on member’s decisions on GRI and the acceptance of secondary findings.

### Study context

The Ethical, Legal and Social Implications (ELSI) of genomics research in the African population project began in 2020 and is ongoing as a dual collaboration between study sites in Nigeria and Ghana. These projects were developed in response to the NIH’s call to support research aimed at the “*return of incidental findings about overall health from clinical and non-clinical data (e.g., genome-wide omics data) produced in studies that focus on dental, oral and craniofacial phenotypes*” [38]. The outcome of large-scale sequencing efforts, including Whole-genome sequencing (WGS), includes secondary findings (SFs), which are out of the scope of the research but are of potential health or reproductive importance. Even though extensive research has been conducted on secondary findings, very little is known about it in minority populations of non-European ancestry. The ELSI project thus presents the opportunity to assess novel aspects of the debate regarding the return of secondary findings in a genomics study, particularly around the comfort and ability of a range of medical and dental providers to return SFs in a region with a weaker genetics infrastructure and with clinicians who likely have less familiarity with genetic testing.

The first aim of the ELSI project explored the rate of identification of actionable SFs in an African cleft lip (with or without cleft palate) cohort using the ACMG SFv3.0 gene list. Actionable pathogenic/ likely pathogenic variants were seen in 2.3% (9/390) of the subjects, a frequency higher than ~ 1% reported for diverse ethnicities [39]. Reduced disease burden and increased understanding of the prevalence and effects of actionable genetic variations in diverse populations are among the major benefits of early risk detection. The second aim evaluated the comfort level, type of expertise and information necessary to return SFs in healthcare providers (HCPs), this is in addition to the interests of parents of children with cleft abnormalities on receiving SFs and their ability to act on it. Very few (1.6%) of the HCPs reported an expert understanding of when and how to incorporate genomic medicine into practice, while 20.21% supported the return of only clinically actionable findings to patients. About 95.4% of patients were willing to receive all the information from genetic testing (including SFs), while the majority cited physicians as their primary information source (64%).

Several barriers were described by providers including limited genetic knowledge, unavailability of patient education resources and uncertain clinical utility. Despite the strong desire expressed by most of the parents regarding the receipts of SFs willingness, the limited access to genetic information remained a major barrier to the return of results in this cohort. Parents also highlighted the community as a significant source of support as they try to navigate testing outcomes with their families. This is quite important in a setting that lacks genetic counsellors who are trained to provide this service and limited genetic knowledge in the HCP as shown in this study. Thus, the third aim of the ELSI project focuses on the role of community gatekeepers in communicating genetic risk information and their willingness to be stakeholders in the genomic testing research process. Gatekeepers are influential in research studies in Africa; by investigating the perception of these essential pillars of the community, this study aims to develop methods to enlist them to help improve decision-making as it concerns participation in genetic testing, improving the perceived benefits, and deciding on the best available management options.

## Materials and methods

### Participant recruitment

We utilized a qualitative research approach via FGDs to describe the perceptions of community gatekeepers regarding GRI in the context OFCs in a sub-Saharan African setting. When conducting research in ethnically diverse populations on a subject on which limited information exists, the qualitative research approach has been recommended [40]. Furthermore, it provides an avenue to optimally engage participants as they offer significant insight into beliefs and experiences while facilitating a thorough appreciation of the concerns around a particular subject [41, 42]. This study focused on community, ethnic, religious, and traditional leaders in Nigeria. Participants who were eligible for the FGDs were adults aged 18 years and older who could either be male or female; Nigerian citizens or residents for a least five years; actively involved in a leadership role (a Christian or Muslim cleric or traditional/community leader) and held a leadership role in their local and religious community in the past two years.

Participant sampling and recruitment followed the organizational structures of each group. Participants were selected via purposive sampling and represented members of the main religious denominations (Christianity and Islam), traditional leaders from the three major tribes (Hausa, Yoruba and Igbo), community leaders and traditional birth attendants. Building on the existing relationship between these groups and the community health department at the College of Medicine, University of Lagos, initial engagement occurred via introductory meetings and presentations to selected churches, mosques, traditional leaders and communities to identify with eligible participants, explain the purpose of the research and seek their support in participation. To promote gender equality and social inclusion and ensure that the responses reflect a range of characteristics of community influencers unaffected by the effect of prevailing paternalism in some groups [43], conscious efforts were made to recruit female leaders, such as female community leaders and women leaders from religious organizations.

### Focus groups

The gatekeepers were engaged in the local communities and via the community health department of the College of Medicine, University of Lagos, after successful community entry [44, 45]. Following the community approach component of the CICP model of CE in genomics research for indigenous communities developed by Ogunrin et al., the community entry process through the interaction with designated leaders in the community served to recognize the position and roles of the gatekeepers, create awareness and gain their support and facilitate the engagement process [29].

A total of 25 FGD sessions: (8 each for Christian & Islamic Leaders), 2 for community leaders,4 for traditional birth attendants (TBAs) and 3 for ethnic leaders (1 Hausa, 1 Yoruba and 1 Igbo) were conducted with 214 gatekeepers across different communities in Lagos, Nigeria from October to December 2021. Each session of FGD involved 8 to 12 people, and sessions lasted 1.5 –2 h. The number of FGD sessions conducted in this study was determined by saturation during data analysis, ensuring a comprehensive exploration of themes until no new insights emerged. Furthermore, the research team, following repeated engagement, reached a consensus on when conducting new FGDs may yield no further information.

We utilized a semi-structured questionnaire to guide the collection of information on participants’ views on GRI and OFC experience. Each group discussion was professionally moderated by a facilitator and a note taker. The FGD guide was developed from literature reviews and consultations with experts in the field of genomic research from the College of Medicine, University of Lagos and the University of Iowa (Supplementary material). A pilot testing was conducted on a small cohort of the intended participants who were not included in the final study and feedback obtained was used to improve the quality of the discussion guide. To ensure ease of communication and understanding, the guide was translated into the three major tribal languages (Hausa, Yoruba, and Igbo).

The FGD guide focused on two main topics: (1) the perceptions of leaders about their members receiving GRI and (2) their duties to act in their members’ best interests when choosing which results to return following genome sequencing. Two researchers coded transcripts from the audio recordings, and emerging themes were discussed with the wider research team. To ensure participant anonymity, all respondents were assigned identification numbers. The codes from the thematic analysis were obtained via a deductive-inductive approach. The principle of saturation played a crucial role in ensuring the accuracy and reliability of the data obtained from the FGDs in this study. Our objective was to reach a point where there were no further insights or themes arising from subsequent FGDs, signifying that we had thoroughly examined the breadth and depth of participants’ experiences concerning the genomic risk information. By methodically evaluating saturation during the data analysis stage, we could definitively ascertain when data collection may be stopped, indicating that we had reached a level of data adequacy that boosts the credibility and reliability of our study’s results. Additionally, audio recordings were transcribed verbatim, as this enhances data rigor and accuracy [46–48].

Theme mapping was guided by the social capital theory (SCT), a social science concept that states that “*social relationships are resources that can lead to the development and accumulation of human capital*” [49]. SCT plays an important role in health promotion because its application to health information can provide an explanation of how social networks impact the understanding and utilization of health-related information [50]. Harnessing social capital can be beneficial in health communication and intervention efforts, as it considers the social environment in which individuals make health-related decisions and underscores the significance of community relationships in fostering positive health outcomes [51–53]. The analysis was done manually and cross-referenced using NVivo 12 to ensure rigor and reproducibility.

## Results

**Fig. 1:**
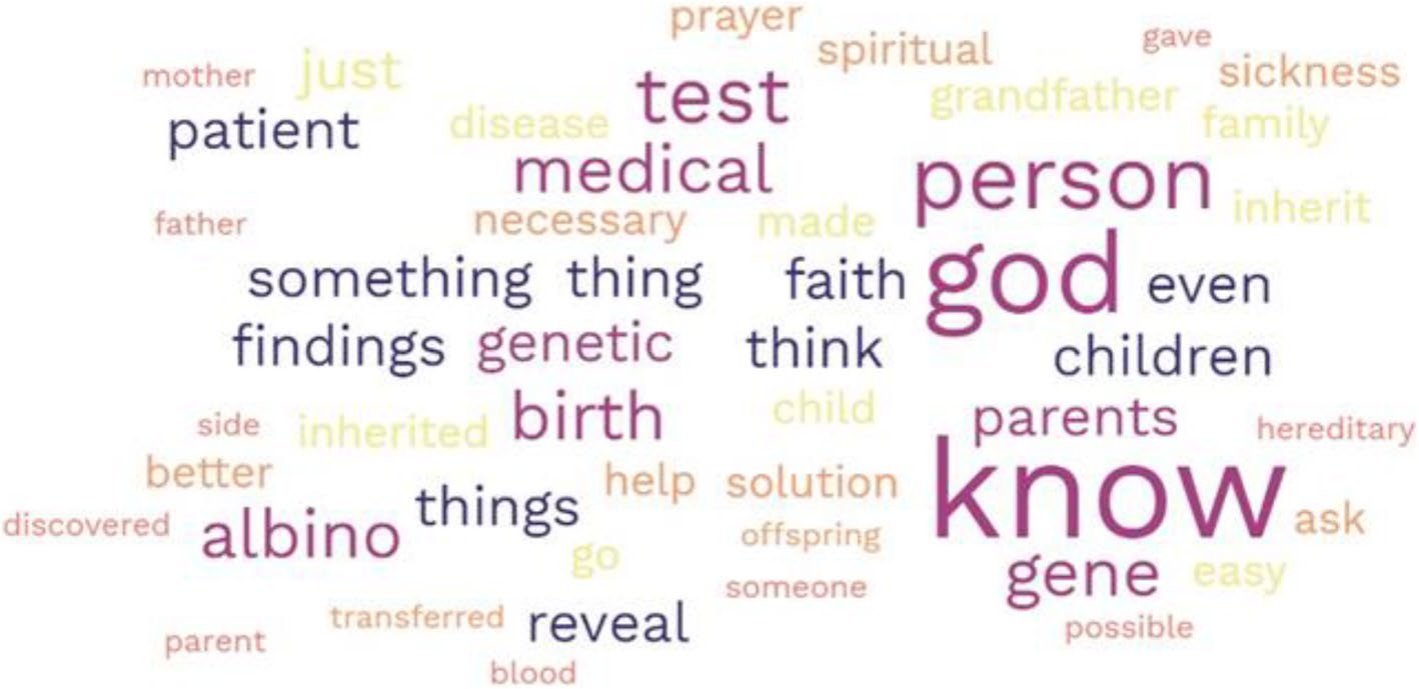
Word cloud figure containing word. The visual representation of the responses obtained following the discussions representing the most frequently used words

A total of 214 gatekeepers participated in the FGDs (Table 1). Participants were recruited from the suburbs of Lagos, a city in Southwestern Nigeria, and they represented the major religious organizations and ethnic identities. Most of the participants were males constituting 74.3% of the study participants. Most (91%) participants self-identified as being of Yoruba ethnicity. The mean age of the participants was 51.6 ± 12.7 yrs. Participants’ perspectives were examined in specific contexts: familiarity with genetics, the role of genetics in health and diseases, views about genetic screening, opinions about receiving secondary findings from testing and concerns about receiving such findings. The majority of the participants (~ 75%) had a positive disposition towards genomic information and the role that it may play in disease prevention. The visual representation of the responses obtained following the discussions is presented in detail in Fig. 1 and further explained in the prominent themes that occurred in this study. After conducting thematic analysis, three overarching themes emerged: knowledge, beliefs and willingness to act.

**Table 1:** Group characteristics

**Group**	**Number of participants**	**Number of women**	**Age range**	**Average age in years (Mean + SD)**
**Religious**				
**Christian**	61	2	35–76	56.3 ± 10.3
**Islamic**	78	22	18–78	48.9 ± 12.5
**Ethnic**	27	8	29–80	50.9 ± 13.0
**Community**	16	1	32–78	59.7 ± 15.3
^a^**TBA**	32	22	21–68	45.6 ± 10.2
**Total**	**214**	**59**		

^a^*TBA* Traditional Birth Attendants

### Theme 1: Knowledge

#### Genomic awareness of community gatekeepers

Most of the community gatekeepers who participated in the discussions showed some form of knowledge and a positive awareness of genetic terms. Also, some have had personal experiences with genetic conditions within their families, communities or congregation members. These opinions were prompted by the questions below.

1. Have you ever heard of the word “gene” or genetics? What do you understand by the words “gene” and/or “genetics” or What comes to your mind when you hear the words “gene” and/or “genetics”?
2. Do you believe that “genes” can be responsible for certain diseases in the human body which can run in families? Or which can be transferred from parent to child OR from generation to generation?

Select excerpts from participant responses are presented below verbatim.

“Gene is the substance contained in the DNA of a person and is transferred from parents to offspring, so it is hereditary, and genetics is the study of gene and genetic variations”- P9CRLGRP5.

“When we talk about genes, we are talking about hereditary that can be traced to either the paternal side or the maternal side”- P5YCLGRP1.

“What I understand by gene is like a trait inherited from parents by the children before and after birth”- P1HL.

“Gene is what you inherit from your parent or your grandfather or grandmother” - P7MWGRP2.

“I have heard but can’t explain it well”- P6YTR.

Participants also believed that diseases and traits could be passed down to the child from the parents and were able to provide examples of conditions with a genetic cause, such as arthritis, diabetes, rheumatism, cancer, sickle cell, and albinism.

“…. it is likely, for example, diabetes and some other terminal sicknesses; if you trace it, you will see that it is something that operates in the family. The father may have had it; then there’s a likelihood that the child will have it”- P4CRLGRP4.

“…that of cancer and sickle cell, children can inherit from their parents because it runs in the blood. So, the babies form with the blood of such things, so it’s easy to inherit from parents”- P4ICL.

“Like Albino, if the parent gives birth, it is possible that 1 out of 2 offspring will be an albino”- P9MLEpe1.

#### Personal experiences of genetic disorders

Gatekeepers shared diverse opinions based on their experiences with genetic disorders such as rheumatism, albinism, and cleft palate. They also shared their thoughts on the causes of these conditions, and the role faith/prayer can play in countering them.

“The disease in my family, rheumatism, when you get to forty (40), fifty (50) or sixty (60) and you are a woman, you will hear oti fi jo iya lagbaja (she has taken after someone - the mother of so), so I know that in my family, it can be transferred”- P3YTL.

“My grandfather is an Albino, and I also have a brother that gave birth to an Albino...When we made our findings, we discovered that his grandfather had given birth to an Albino before”-P4YTL.

“Like the Lord says that he is not going to test us more than our faith, so even though the thing is inherited, if we pray to God, due to what he has said and we believe in him, nothing will happen to us”- P5CRLGRP6.

“That is what we are saying as far as ministerial experience is concerned to go back to the medical line, because, without experience in the ministry, we may associate such experiences to spiritual attack or living against the will of God”- P4CRLGRP6.

“There are some families who believe that they have forbidden things, so anybody that trespasses or engages in those forbidden things, such diseases Oro-facial cleft can happen to such person”- P6CRLGRP6.

### Theme 2: Belief

#### Community gatekeeper’s attitudes toward genetic screening/testing

Gatekeepers appreciated the value of genetic screening and believed that genetic screening/testing has a role in helping individuals to live a healthier life, such as disease prevention, empowerment to make healthier choices, knowing the direction of prayer and preserving the family unit or structure. These opinions were prompted by the question below.

1. What are your views on genetic screening/testing? Do you think it holds any value? And if yes, what do you think is the value of conducting such screening tests?

Select excerpts from participant responses are presented below verbatim.

“The genetic test holds value because even in faith, if you know what happens, you can pray it out of your life, but something needs to tell you, and this test will reveal it has value” -P4CRL.

“When you get tested, the screening tells you something, and then you are prepared to avert medically”-P6CRLGRP3.

“Do you know why you should go for a test? So that we can know the way to manage it even if it is incurable.”-P4CRLGRP3.

“It’s necessary to check even before you get married, to check your genes, because these types of things have broken many homes.”- P2ICL.

“So that the person will know the medication to take care of it”- P1MLGRP3.

“I think it is very, very important, especially in this modern era where we are in where there is advancement in medical and technological sciences” - P6CRLGRP4.

“In my church, we can ask you, people, to come, and you can carry out the test because it will help to reduce premature death when you know the type of genes that you have” - P5CRLGRP3.

#### Community gatekeepers’ attitude towards genomic risk information

In addition to the diverse socio-demographic characteristics, gatekeepers’ perceptions towards receiving genomic risk information and their roles in assisting members with testing outcomes also differ. While some were ready to take proactive roles in seeking solutions (preventive or curative) either medically or through faith, others felt demoralized and would even reject such results. These opinions were prompted by the questions below.

1. What is your opinion about receiving secondary findings from genomic test? Do you think it is better to know or not to know of such findings?
2. If your patient or someone in your community undertook a genetic screening test and the test revealed some secondary findings, and that person or family sought your advice, how would you advise such a person/family?
3. Do you have any concerns about relaying secondary genomic findings back to patients? If yes, what are your concerns about this, considering that such findings may not be related to the original reason for the test?

Select excerpts from participant responses are presented below verbatim.

“I will look for ways of asking for prevention because for every problem there is always a solution, so what I will be doing is to see how it can be managed if possible or take precautions so that my siblings and children wouldn’t go through the same experiences”-P6CRLGRP4.

“I will find the solution to it in any way, whether medical or spiritual”-P2YTL.

“Well, medically, I will ask for advice and some drugs that I will be using for the disease”-P3CRLGRP6.

“I don’t think you can feel bad about something you cannot control; if God has created you and any medical occurrence is explained to you, you should accept your faith”-P1CRLGRP1.

“I think with such a condition, you should tackle it with spiritual power; I mean, through prayer, God can heal that sickness because he promised that whatever we ask from him, he is able to answer us, and there is nothing difficult for God to do so. Therefore, such sickness needs prayer; I don’t think that it is by mistake that the child has it; it may be an attack from the devil or powers of the darkness. To overcome such, you need to tackle it with prayer, and God is able to heal any sickness”-P2CRLGRP1.

“If anybody comes to me about what you’ve just said, I will tell them to reject it that it is not their portion…. because it is not from God”-P1CRLGRP6.

Gatekeepers also showed varying attitudes towards receiving secondary findings from genomic testing.

“If a person comes findings that secondary findings were discovered, the best way is to encourage the person to strengthen his faith and let the person knows that it is not a death sentence. It is just a discovery which means that every ailment has treatment or solution so encourage the person if there is any medical thing they can still do”- P3CRLGRP1.

“It is better not to know because, especially as a man of God, you shouldn’t be looking for what is not necessary. In the Book of 2 Samuel 24, King David was counting the children of Israel for what God did not instruct him to be counting. He just counted, finding what is not in your body; it is not necessary”- P4CRLGRP2.

#### Opinions on relaying secondary genomic findings

Although there was an overwhelmingly positive attitude towards receiving genomic information, gatekeepers have varying opinions on the mode of relaying secondary genomic findings. These include following professional guidelines, involving community members, returning secondary findings with proper patient counselling, and maintaining utmost confidentially. Excerpts from their responses regarding the return of secondary genomic findings are presented below:

“Present it normally according to the ethics of your profession”-P1CRLGRP5.

“….so, the doctor who is involved should seek experienced doctors or possibly pastors of the involved patient to package an excellent information which will suit the person’s mind in handling the situation so that it would not lead to hazard situation”-P4CRLGRP5.

“It is good to reveal the result, but the doctor that will reveal it must be someone sensitive enough….so, anyone that wants to counsel must be patient and give a soft word”-P4TBAGRP3.

“It is good to reveal such information to the patient so that he can know what is wrong with him. Just as P3 has said, some don’t want anyone to know the nature of the disease in public, but when they know it secretly, they can take care of it”-P4 TBAGRP3.

### Theme 3: Willingness to act

#### Gatekeeper’s role and opinion in supporting community members

When asked what their role in supporting members involves, the participants expressed their willingness to provide support via different means such as counselling, prayer or offering financial support. These opinions were prompted by the question below.

1. As a TBA/community leader, would you be willing to give your support or otherwise to your members or someone close to you to learn or receive any secondary findings as a result of genomic screening? (If yes, what kind of support do you think you can/will give; if no, please explain why).

Select excerpts from participant responses are presented below verbatim.

“It is a job made easy; I seriously support carrying out genetic testing in anything. You want to do it because medicine has made life easy for pastors”-P1CRL.

“Without any explanation, if there is need for me to counsel or refer, I will do it because it will help you or it will help the individual to better themselves “-P5CRLGRP1.

“I will be curious to know and will do the test and find out the result and the normal thing to be done”-P5CRLGRP2.

“I will not object to doctors’ recommendation for genetic screening. Because he is the expert, he is the one seeing me; he knows it better than myself” -P6CRLGRP2.

“What I will give him advice, and if he doesn’t have the money, I will support him and take him to the hospital”-P9HL.

#### Community gatekeepers’ experiences with orofacial clefts

Many participants had encountered OFCs before this study either as they provided care (TBAs) or within their congregation (religious leaders).

“I had a patient who gave birth to a baby that had cleft lip and palate, and when she had the second child, it was the same thing. I had to do my findings before discovering that the mother has it and she also inherited it from her father”-P6TBA.

“I have seen it before; I have seen that of a child and also a grown-up person”. -P10 MLGRP2

Also, their opinion on the possible causes of OFCs ranged from malnutrition, the use of wrong medication and maternal illness during pregnancy to cultural beliefs such as taking late night walks or engaging in forbidden acts.

“In addendum to what he has said earlier on there are some families who have this belief that they have forbidden things so anybody that trespasses or engages in those forbidden things such Oro-facial cleft can happen to such person”- P6CRLGRP6.

“Sometimes early pregnancy, when you are not aware that you are pregnant, you start taking some drugs, which is not good for the baby. It can deform the baby.”-P9 ICL

However, mixed reactions were recorded about the genetic underpinnings of OFCs. While some participants believed that OFCs can be transferred across generations, others disagreed while some held off commenting because they had never experienced OFCs.

“Medically it is believed that since the gene is a trait from the source, from the parent to the offspring, then it is believed that it can transmit medically from the source that is the parent to the offspring upon the generation if not cured o rprevented”-P5 ML GRP1.

“I have seen Oro-facial cleft and I don’t feel that it is transferable I just felt it is just like a mutation that could have happened more like Down Syndrome thing and it can it be as a result of some other things so for me I can’t say that it is A genetic thing rather it could be a mutation that could have happened due to somefactors”-P4CRL GRP 4.

“I will be neutral into it, and I will rather say I believe or not. Because I have never experienced that”-P5CRL GRP 4.

For participants who had encountered OFCs or previous contact with cleft affected families, they were able to act in their role as gatekeepers to provide moral, spiritual, emotional, and financial support in addition to advice about where to find them cleft care services.

“When I was told and I saw the baby, I was also confused on what to do… I prayed, fortunately we have a medical doctor in the church, he also worked in LUTH here”- P2CRLGRP2.

“…another one happened in my own clinic; all my other staff ran away but I stood by the mother”- P2TBAGRP2.

“I have encounter with someone that has OFC, he was shy and because of stigmatization so I happened to have an encounter with medical doctor, and they said that there is free surgery for such cases. So, I let him understand that this is what is happening, he said he never knew. lo and behold he went for that surgery, and it was well placed now, when you look at him you will never know that he has such a thing”-P6CRL GRP6

“The support we will give them is that They should visit the hospital and if there are not financially buoyant the congregation can contribute money forthem”-P2MLGRP2.

## Discussion

The current study aimed to describe the opinions of gatekeepers in African communities on genomic risk information in the context of oro-facial clefts, their opinions on what role they could play in supporting their members following the return of genomic risk information and determine the best strategy for preparing them to help disseminate, support and encourage the appropriate utilization of genomic risk information in their communities. This project uniquely contributes to the ethical, legal, and social implications (ELSI) discourse in African genomics research by exploring how socio-cultural and religious values and structures influence the interest, interpretation, understanding and utility of genetic and genomic services [54].

Overall, the attitude towards genomic information was largely positive. Participants believed that genetic testing might play a role in preventing adverse health outcomes. This contrasts with the report by Uebergang et al., where participants had no previous genomic experience and were only aware of heredity-related issues [17]. However, Naidoo et al. reported outcomes similar to our study in their research, which explored stakeholder perception toward the use of predictive genetic testing in a South African community [55]. Additionally, gatekeepers showed varying degrees of genomic knowledge and awareness based on the data gathered from the self-reported information provided during the FGDs. This level of awareness can be improved upon by providing them with the needed training to help improve the quality of messages disseminated in the community. A similar approach to empowering community leaders has been reported in Africa [4, 15]. Although majority had encounter cleft affected individuals and families at some point in their role as a gatekeeper, superstitions, and cultural beliefs about the etiology of OFCs persist and suggests the need for improving the literacy and awareness about OFCs as a broader aspect of understanding genomic risk information in the study context.

Gatekeepers also believed genomic testing was valuable because it could empower patients with useful information while testing outcomes could help doctors better manage their condition. Akinyemi et al. reported similar findings in a West African stroke cohort [56]. Participants were also willing to act as “representatives” of the research and clinical enterprise in their communities. One of the barriers to community engagement in genomic research remains how to explain what the study entails in a manner that promotes access and positive engagement. The willingness of participants in this study to act as pillars of support is a place to start building long-term structures in these communities following the establishment of beneficial collaborations. In this study context, the connections and networks built within the social environment of cleft-affected families, such as that with the different categories of gatekeepers, can be harnessed to improve the understanding and utilization of GRI in those affected, their families and their communities. Study findings showed that CGs were willing to use their social influence to improve the understanding of GRI in their communities but needed to be formally trained to serve in these roles. They therefore proposed several means –– seminars, workshops, and social groups (community / religious gatherings) to collaborate with researchers and improve their knowledge of genomic information. Engaging and training local leaders to promote genetic literacy in communities is not new. Studies continue to show the importance of grass root inclusion, i.e., acting as community ambassadors, participating in the institutional review process and the unique characteristic of leaders to understand issues affecting their constituents towards improving the understanding of genomic information [4, 57]. Study participants believed that a history of a genetic condition and advice from medical personnel and religious leaders were enough motivators to seek genetic or genomic testing.

The advances in genetic and genomic technologies are likely to widen existing racial and ethnic disparities in health, particularly in racial minorities who are underrepresented in large-scale genomic efforts, thus a need to develop strategies aimed at improving awareness and effective communication to bridge this gap [58]. In the context of African genomics research, low genomics literacy persists among the public and healthcare providers [4, 16, 59]. This is in addition to a lack of support for patients and mistrust of the research enterprise. Thus, engaging communities can help unravel the complexities of conducting genomic studies by providing an avenue to improve genomic literacy, return study findings, and establish a trusting participant-researcher relationships [25, 29, 60].

Improving access to genomics services is being led by global organizations such as the World Health Organization (WHO). A recent report from the WHO Science Council outlines areas to promote the implementation of genomic services and ensure benefits for all across four main themes, namely “promotion through advocacy, implementation of genomic methodologies, collaboration among entities engaged in genomics, and ethical, legal, and social issues” [61]. The report also stressed the need to increase public genomic awareness and understanding to build trust [61].

Research has shown that gatekeepers are uniquely positioned to recognize, comprehend, and deal with the contextual factors contributing to stigma in their societies [14]. Furthermore, Kimotho et al. showed that increasing public awareness of cleft could help address the stigma associated with this condition [62]. Thus, the focus on gatekeepers in this study could help address the double ethical issue of stigmatization associated with OFC and genomic findings in African populations. The public would profit from applying genomics to their health if they had a clear understanding of fundamental genetics concepts and could obtain, recognize, and use reliable information [61, 63]. In developing health literacy interventions in resource-limited settings, it is essential to explore inclusive methods that reflect cultural and linguistic diversity, local context and accommodate the full range of cognitive and literary skills [64]. Moreso, interventions aimed at promoting genomic literacy in participants could be more effective where complementary interventions are created for stakeholders such as providers, religious leaders, and other influential groups who can foster a supportive environment in the community.

## Conclusions

Our findings demonstrated how establishing relationships with communities through influential channels can contribute to the global ethical debate in genomics research. Lessons learnt could help develop appropriate community engagement strategies to manage conflicting ideologies surrounding genomic information for individuals and different communities while allowing for a more equitable utilization of advances in genomics research in minority populations.

### Limitations

Firstly, research has shown that educational attainment can affect how individuals understand genomic risk information [65]. Although we recruited a diverse sample of community gatekeepers, our analysis did not include comparisons based on participants educational attainment or other important socio-demographic variables such as occupation. This constitutes an important study limitation and future research will be needed to examine the impact of these socio-demographic variables on participants’ understanding of genomic risk information. Secondly, the study site was in an urban city in West Africa; hence our findings may fail to capture possible variations in the perception of genomic risk information due to the cultural and religious diversity across the African continent. Therefore, we advise that the results be interpreted cautiously. However, it provides a foundation to explore further the role of community gatekeepers in African genomics research. Thirdly, other important community gatekeeper groups with the potential to enrich the perspectives reported such as traditional/ spiritual healers (i.e. Babalawo’s, Dibia’s, Boka’s etc.) were not included in this study, due to the crucial role they play in the understanding of health-related information, particularly for individuals living in remote/underserved areas, further studies that improves the representation in community gatekeeper groups are recommended to capture these important opinions. Finally, this is the first ELSI study to explore the role of gatekeepers in managing genomic risk information in the context of OFCs in a sub-Saharan African Population. Further research on the socio-cultural uniqueness of the African population is needed to capture opinions that better represent the diversity across the continent.

### Supplementary Information

**Supplementary material 1.**

## Data Availability

The datasets generated and/or analyzed during the current study are available from the corresponding author on reasonable request since these data are qualitative and stored in a recorder and transcribed into text using NVIVO and manually.
