# Supplementary material for: Perceptions and beliefs of community gatekeepers about genomic risk information in African cleft research
Source: BMC Public Health. 2024 Feb 17;24:507. doi: 10.1186/s12889-024-17987-z (PMC10873930; doi:10.1186/s12889-024-17987-z)
Supplement: Supplementary file 1 — Supplementary material 1. [file 12889_2024_17987_MOESM1_ESM.docx]

**Focus Group Discussion Guide for Community Gatekeepers’ Perceptions of Secondary Genomic Research Findings**

**Introduction**

- Introduce yourself- Mention your name and that you’re a member of a research team from the College of Medicine, University of Lagos
- Explain the nature and purpose of the study
- Explain the fact that there are no right or wrong answers; it is their views/opinions that are needed and not the opinions of any researcher. Encourage them to “be free” to express their minds.
- Seek their consent- written/verbal informed consent and let them know that the discussions will be recorded and why
- Collect their basic socio-demographic information and assign codes
- Inform them of the ground rules- i.e. they need to mention their codes before they speak, put phones on silent etc.
- Inform them that the discussion will take about 2 hours or so.
- Both moderator and note taker are to be familiar with the FGD guide BEFORE the event.

*Note-taker to take additional pen and writing materials. Ensure you have your voice recorder checked as well as a back-up before the discussion.*

We are researching to obtain the opinions of community, religious and ethnic leaders on secondary genomic research findings and to learn their decisional preferences about genomic test results. Our discussions will focus on two themes: Your feelings about receiving genomic risk information, your opinions as a leader and your perceived role in weighing on your members decisions on genomic risk information and the role of secondary findings.

Because of your leadership role in the [community], you have been selected to voice your views to achieve this purpose. We therefore humbly seek your participation in this research. We assure you that any information you provide will be treated with strict confidentiality, and your responses will be anonymized.

Please feel free to share your heartfelt opinions on all issues raised in the course of this discussion. There are no right or wrong answers. Neither your name nor the name of your community will be mentioned in this research, and all your responses are confidential.

Please accept our gratitude in advance for your time and attention.

Do you possibly have any questions about the study before we start the session?

**Questions**

1. Have you ever heard of the word “gene” or genetics? What do you understand by the words “gene” and/or “genetics” or What comes to your mind when you hear the words “gene” and/or “genetics.”
2. Do you believe that “genes” can be responsible for certain diseases in the human body which can run in families? Or which can be transferred from parent to child OR from generation to generation?

**Probe:** If yes, give examples or share experiences of genetic disorders which you think could be inherited in the family.

**Probe:** To what extent do you believe genes contribute to health and disease?

1. Show picture of Oro-facial Cleft. Do you think OFC runs in families? And why? Or Why not?

Mention some of the genetic conditions below or use some of the examples of the genetic conditions mentioned by the participants. *Examples could be; Albinism, Down syndrome, Cleft lip and palate, Epilepsy, Diabetes, Sickle cell anemia, Heart disease, High blood pressure, Breast cancer, Colon cancer etc.*

1. Based on the examples of genetic conditions mentioned, How would you feel if you (or someone close to you) was told that a genetic condition such as any of these runs in your own family or could manifest in future in you or any of your family members?

*Genetic screening/testing is a test you can take to check your genes for diseases that may run in you and can be passed on to your family members.*

1. What are your views of genetic screening/testing? Do you think it holds any value? And If YES, what do you think is the value of conducting such screening tests?

**Probe:** Are there any reasons that might motivate you to undergo a personal genetic screening test? If YES, what are such reasons?

**Probe:** Are there any reasons that might make you advice someone you know to undergo a genetic screening test? If YES, what are such reasons?

**Probe:** How would you feel and what would you do if you [*or someone close to you*] was asked by your doctor to undertake personal genomic screening or testing?

**Probe:** What is your view of genomic *pre-conception carrier screening*? Is genomic pre-conception carrier screening worth knowing?

**Probe:** If YES, Why do you think so?

**Probe:** If , Why do you think so?

**Probe:** What if *one of your patients/community members* was asked by a doctor to undertake personal genomic testing and sought your advice? How would you handle it? What would you tell him/her?

**Probe:** How about for genomic pre-conception carrier screening. What would be your advice to that person?

**Probe:** How about if the genomic testing would allow prevention of manifestation of a disorder in the future OR if it could avoid passing on the genetic conditions to offspring OR provide information on prevention for your offspring.

**Probe:** How about if the personal genomic testing was for a disease that cannot be cured

**Probe:** How about if the personal genomic testing was for a disease that cannot be managed with orthodox medicine

A genetic screening test is a test that checks a person to see if they have a “gene” for a specific disease.

**Probe:** How about if the personal genomic testing was for a disease that cannot be prevented?

*Secondary findings are information about genetic diseases that are found out during a genetic screening test outside of the original purpose of that test. For example, if someone in the family runs a genetic screening for a disease like oro-facial cleft lip, they may [secondaryly] find out on screening that the person has a gene for another disease like albinism or Down’s syndrome or even high blood pressure.*

1. What is your opinion about receiving secondary findings from genomic test? Do you think it is better to know or not to know of such findings?
2. **Probe:** Do you have any concerns about relaying secondary genomic findings back to patients? if, YES, what are your concerns about this, considering that such secondary findings may not be related to the original reason for the test?
3. If your patient or someone in your community undertook a genetic screening test and the test revealed some secondary findings, and that person or family sought for your advice, How would you advice such a person/family?
4. As a community leader, would you be willing to give your support or otherwise to your members or someone close to you to learn or receive any secondary findings as a result of genomic screening?

Probe: If YES, what kind of support do you think you can/will give

Probe: If NO, Please explain why

1. Do you think there are any barriers to genetic screening and returning of results and secondary findings in particular? What sort of barriers? Please mention them and elaborate
2. Do you have any other comments to make on this topic of discussion?

.

**Thank you for your time and for agreeing to be part of this.**
